# Expertise and the representation of space

**DOI:** 10.3389/fpsyg.2014.00270

**Published:** 2014-04-03

**Authors:** Michael H. Connors, Guillermo Campitelli

**Affiliations:** ^1^ARC Centre of Excellence in Cognition and its Disorders, Department of Cognitive Science, Macquarie UniversitySydney, NSW, Australia; ^2^Dementia Collaborative Research Centre, School of Psychiatry, University of New South WalesSydney, NSW, Australia; ^3^School of Psychology and Social Science, Edith Cowan UniversityPerth, WA, Australia

**Keywords:** big data, chess, chess expertise, chunks, expertise, decision making, methodology

Across many domains, experts make decisions based on the spatial relationships of objects within an environment. Firefighters, for example, need to evaluate the fire in front of them, radiologists the medical scan, and chess players the position on the chess board before making a decision. In order to be effective, experts need to assess these spatial relationships quickly and despite possible uncertainty. This ability is facilitated by cognitive processes. According to the so-called chunking and template theories, this expertise is made possible by pattern recognition (Gobet and Simon, 1996; Gobet, 1997). Based on their experience and practice, experts store in their long-term memory meaningful configurations of elements (i.e., patterns—chunks or templates) in their domain of expertise. These patterns are associated with typical decisions and strategies. Thus, when an expert faces a situation in their domain of expertise, they are able to rapidly recognize patterns, which, in turn, prompt them to consider decisions or strategies that have been effective in previous situations. This allows experts to make superior decisions to novices who are limited to a real-time search through a large number of possible options (Feltovich et al., 2006; Gobet and Charness, 2006; Connors et al., 2011).

Leone et al.'s (2014) elegant study adds to existing evidence for this account. In particular, Leone et al. demonstrate that experts' representation of space in their domain of expertise is qualitatively different to that of novices. Leone et al. focused on chess. Chess has the advantage of being a constrained task environment with relatively high ecological validity to other domains. Chess also has the advantage of a precise measure of expertise in the form of a numerical rating that is assigned to each player on the basis of their performance against their opponents. For these reasons, a large amount of expertise research has examined chess (Gobet and Charness, 2006). For Leone et al.'s study, chess has the additional advantage of having large amounts of data available: Leone et al. examined well over 175,000 games drawn from an internet chess server.

Given experts' pattern recognition ability, Leone et al. predicted that experts should make moves that were best for the position at hand and so not be constrained by the mere physical distance between objects. In contrast, novices would be more likely to make moves based on spatial proximity to previous moves due to their relative lack of pattern recognition and their need to conserve cognitive resources. Leone et al. made four specific hypotheses. First, the authors predicted that novices should make moves that are closer to their or their opponent's previous move. To test this, they developed a novel methodology in which they calculated the distance between a player's move and their previous move, and the distance between a player's move and their opponent's previous move. Second, the authors predicted that novice players would be more likely to move the same piece multiple times. To test this, they examined the frequency with which players moved the same piece more than once on consecutive turns. Third, the authors predicted that novices would be more likely to simplify the position by exchanging pieces (capturing an opponent's piece when the opponent can recapture their own piece), thereby reducing the cognitive load of dealing with large numbers of pieces. To test this, they examined the rate at which pieces were removed from the board according to the number of moves in the game. Finally, the authors predicted that novices would be less likely to keep with general strategic principles (e.g., knights are more effective when centralized) than experts. To test this, they examined the frequency with which pieces were placed in suitable parts of the board (e.g., knights in central regions).

Leone et al. found evidence for all four hypotheses. First, novices were more likely to move pieces that were closer to their or their opponent's previous move than experts. Second, novices were more likely to move the same piece on consecutive turns than experts. Third, novices were more likely to simplify positions than experts. Finally, novices were less likely to keep to general strategic principles than experts. Although the relative size of the effects were small, Leone et al. demonstrated the robustness of the effects across very large datasets and across games of different durations. Similar differences between experts and novices were evident regardless of whether players had a total of 3, 5, or 15 min each.

Although the authors did not directly assess the quality of the players' decisions, their work provides evidence that experts base their decision on what is most meaningful in a position, rather than being limited by spatial proximity. As Leone et al. note, these findings are consistent with chunking and template models of expertise as the differences between experts and novices exist at very short time limits, when searching through various options is not possible. The findings are also likely to generalize to a wide range of other domains of expertise. In addition to revealing these effects, the work is very important because it develops novel methodologies for assessing spatial relationships in chess positions and across large datasets. Indeed, Leone et al.'s use of big data to test specific hypotheses about cognition is particularly innovative. In future, these methods might provide further insight into how experts interpret and represent space.

## Conflict of interest statement

The authors declare that the research was conducted in the absence of any commercial or financial relationships that could be construed as a potential conflict of interest.
